# Loss of CYLD on Chromosome 16q Impairs Homologous Recombination and Genomic Stability Through TIRR Degradation

**DOI:** 10.1002/advs.77298

**Published:** 2026-08-21

**Authors:** Mingming Lu, Jialu Kang, Qi Ye, Zixi Wang, Lei Li, Yuzeshi Lei, Leihong Ye, Tianjie Liu, Bin Wang, Tao Liu, Shan Xu, Ke Wang, Yong Zhang, Jian Ma, Lei Li

**Affiliations:** ^1^ Department of Urology The First Affiliated Hospital of Xi'an Jiaotong University Xi'an People's Republic of China; ^2^ Department of Urology The First Affiliated Hospital Zhejiang University School of Medicine Hangzhou People's Republic of China; ^3^ Department of Breast Surgery The First Affiliated Hospital of Xi'an Jiaotong University Xi'an People's Republic of China; ^4^ Department of Surgical Oncology The First Affiliated Hospital of Xi'an Jiaotong University Xi'an People's Republic of China

**Keywords:** CYLD, deubiquitination, DNA damage and repair, targeted therapies

## Abstract

Loss of chromosome 16q is a recurrent genomic alteration in bladder and prostate cancers and is associated with poor clinical outcomes. However, the mechanisms by which 16q loss contributes to tumor progression remain poorly understood. Here, we identify the deubiquitinase CYLD as a major contributor to genomic instability associated with chromosome 16q deletion. Mechanistically, CYLD stabilizes the 53BP1 regulator TIRR by removing K48‐linked polyubiquitin chains, thereby preventing excessive accumulation of 53BP1 at sites of DNA damage and maintaining efficient homologous recombination repair. Loss of CYLD disrupts this regulation, shifting DNA double‐strand break repair toward 53BP1‐dependent non‐homologous end joining, leading to homologous recombination deficiency. Consequently, CYLD‐deficient tumor cells exhibit increased sensitivity to PARP inhibitors. Together, these findings establish CYLD as a critical regulator of DNA double‐strand break repair pathway choice and suggest that CYLD loss, or chromosome 16q deletion, may serve as a biomarker of genomic instability and a predictor of response to PARP inhibitor therapy.

## Graphical abstract

Chromosome 16q loss drives genomic instability through disruption of the CYLD–TIRR–53BP1 axis. CYLD preserves homologous recombination by stabilizing TIRR and limiting 53BP1 accumulation at DNA double‐strand breaks. CYLD deficiency redirects repair toward error‐prone non‐homologous end joining, promotes mutational burden and homologous recombination deficiency, and renders tumor cells vulnerable to PARP inhibitor‐induced synthetic lethality.

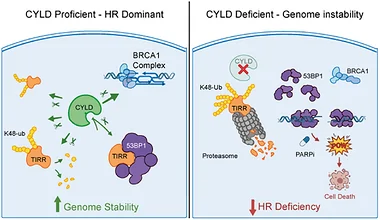

## 1 Introduction

Chromosome 16q loss is among the most prevalent and recurrent genomic alterations across a spectrum of human malignancies, particularly in bladder, prostate, and breast cancers [1, 2, 3]. Large‐scale genomic sequencing has consistently associated 16q deletion with advanced tumor stage, increased metastatic potential, and significantly poorer clinical outcomes [4, 5]. While this locus is known to harbor several tumor suppressors, such as *CDH1*, the specific “driver” genes responsible for the pervasive genomic instability observed in 16q‐deficient tumors remain largely uncharacterized [6, 7]. Given that 16q loss often occurs alongside complex chromosomal rearrangements, identifying the molecular drivers within this region is essential for understanding tumor evolution and uncovering latent therapeutic vulnerabilities.

Genomic instability, a fundamental hallmark of cancer, primarily arises from defects in the DNA damage repair (DDR) machinery [8, 9, 10]. A quintessential example is the deficiency in homologous recombination (HR) repair, which leads to a “BRCA‐ness” phenotype [11, 12, 13]. While the success of Poly (ADP‐ribose) polymerase inhibitors (PARPi) has demonstrated the power of synthetic lethality in *BRCA1/2*‐mutant cancers, clinical responses in *BRCA*‐wildtype tumors remain heterogeneous and often limited by intrinsic resistance [14, 15]. Current diagnostic markers for HR deficiency (HRD) often fail to capture the dynamic proteostatic or epigenetic shifts that can similarly impair DNA repair. Therefore, there is an urgent clinical need to define non‐canonical mechanisms of HRD and identify robust biomarkers beyond *BRCA1/2* to expand the cohort of patients who can benefit from PARPi therapy.

The choice between HR and non‐homologous end joining (NHEJ) is a decisive event in double‐strand break (DSB) repair, largely orchestrated by 53BP1 [16, 17, 18]. By favoring NHEJ and actively restricting DNA end resection, 53BP1 serves as a master regulator of pathway choice [19, 20]. Its activity is strictly modulated by TIRR (Tudor‐interacting repair regulator), which masks the 53BP1 Tudor domain under basal conditions to prevent premature chromatin engagement [21, 22, 23]. We previously demonstrated that DTX3L functions as a key regulator of this axis, highlighting the importance of ubiquitin‐dependent signaling in maintaining TIRR levels [24]. However, the regulatory landscape of this axis remains incomplete. Specifically, whether a dedicated deubiquitinase (DUB) exists to counteract TIRR degradation and dynamically stabilize the HR/NHEJ balance in response to genotoxic stress has remained an open question in the field.

In the present study, we demonstrate that CYLD, a deubiquitinase frequently co‐deleted within the 16q locus, is a critical determinant of the genomic instability associated with 16q deletion. Mechanistically, we show that CYLD directly interacts with and stabilizes TIRR by removing K48‐linked polyubiquitin chains, thereby preventing its proteasomal degradation. Loss of CYLD leads to a rapid decline in TIRR protein levels, causing the uncontrolled accumulation of 53BP1 at DNA damage sites and a subsequent shift toward NHEJ at the expense of HR repair. This CYLD‐driven HRD provides a novel mechanistic explanation for the genomic instability following 16q loss and, crucially, confers hypersensitivity to PARP inhibitors. By establishing the CYLD–TIRR–53BP1 axis as a central arbiter of DSB repair, our findings not only redefine the functional landscape of chromosome 16q but also nominate CYLD loss as a precision biomarker for PARPi responsiveness in a broad range of cancers.

## 2 Results

### 2.1 Chromosome 16q Loss Drives CYLD Downregulation and Genomic Instability in Cancer

**FIGURE 1:**
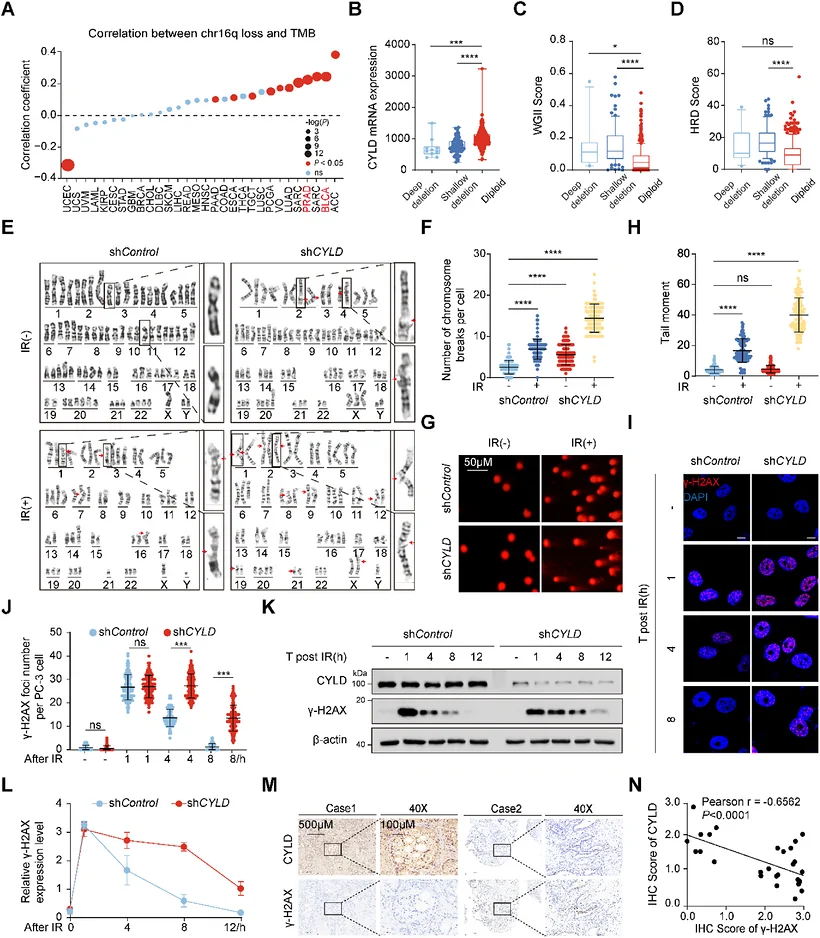
Chromosome 16q loss drives CYLD downregulation and genomic instability in cancer. (A)Analysis of the correlation between chr16q loss and tumor mutational burden (TMB) across TCGA cancer types. Correlation coefficients are shown. Dot size represents−log10(*p* value). Dot color indicates statistical significance (red, ^*^ *p* < 0.05; blue, ns). Spearman's rank correlation tests. (B)Analysis of CYLD mRNA expression across chr16q copy‐number status in prostate cancer samples from TCGA. CYLD mRNA expression is shown as log2(RSEM + 1). Kruskal‐Wallis test followed by Dunn's multiple‐comparisons test; ^***^ *p* < 0.001; ^****^ *p* < 0.0001. (C and D) Analysis of weighted genome integrity index (wGII) score (C) and homologous recombination deficiency (HRD) score (D) across *CYLD* copy‐number status in prostate cancer samples. The box outlines show the 25th and 75th percentiles, and the solid lines show the median value. Means and SDs are represented. Kruskal‐Wallis test followed by Dunn's multiple‐comparisons test; ^*^ *p* < 0. 05; ^***^ *p* < 0.001; ^****^ *P*< 0.0001. (E and F) Control or CYLD knockdown PC‐3 cells were treated with or without IR(2 Gy) for 2 h before being harvested. Cells were harvested for karyotyping, and chromosome breaks in more than 100 cells from three biological replicates in each group were counted and quantified. Representative images were shown in (E), and quantitative data are shown in (F). Means and SDs are represented. Two‐way ANOVA followed by Tukey's multiple‐comparisons test; ^****^ *p* < 0.0001. (G and H) Genomic stability analysis with comet assay in PC‐3 cells. Cells were treated with or without IR(2 Gy) for 2 h before being harvested. Scale bar, 50 µm. Analysis of comet tail shown in (H) using the Comet Assay Software Project. Means and SDs are represented. Two‐way ANOVA followed by Tukey's multiple‐comparisons test; ns, non‐significant; ^****^ *p* < 0.0001. (I and J) Immunofluorescence analysis (I) and quantification (J) of CYLD and γ‐H2AX in Control or CYLD knockdown PC‐3 cells treated with or without IR (6 Gy). Scale bar, 100 µm. Data were shown as the mean ± SD from 20 fields (>100 cells, *n* = 20) from three biological replicates. One‐way ANOVA followed by Tukey's multiple‐comparisons test; ^***^ *p* < 0.001. (K and L) WB analysis of CYLD and γ‐H2AX in whole‐cell lysates of Control or CYLD knockdown PC‐3 cells post IR (6 Gy). (L)Quantification of relative CYLD and γ‐H2AX expression was performed across three biologically independent experiments. Means and SDs are represented. (M)Representative images of IHC staining of CYLD and γ‐H2AX protein from prostate cancer samples (*n* = 28). Scale bar in 10 X fields: 500 µm. Scale bar in 40 X fields: 100 µm. (N)Correlation analysis between CYLD and γ‐H2AX IHC score was shown in tumor tissues. Pearson r = ‐0.6562; *p* < 0.0001.

Chromosome 16q loss is a recurrent genomic alteration observed across multiple cancer types [2, 25, 26]. Analysis of TCGA datasets (https://www.cbioportal.org/) revealed that 16q loss is associated with elevated tumor mutation burden (TMB) across diverse cancers, with particularly strong enrichment in prostate and bladder cancers (Figure 1A), suggesting a role in promoting genomic instability. Consistent with previous genomic analyses and DepMap datasets, we utilized MCF‐7 cells as an independent, chromosome 16q‐deficient cancer model [27]^.^ To identify the functional drivers within this locus, we screened several tumor suppressor genes located on chromosome 16q by restoring their expression. Notably, only the restoration of CYLD significantly increased homologous recombination (HR) reporter activity upon DNA damage (Figure S1A,B). Based on these findings, we next examined the relationship between 16q loss and *CYLD* expression. TCGA data analysis demonstrated that 16q loss is significantly associated with reduced *CYLD* mRNA levels in both prostate and bladder cancers (Figure 1B; Figure S1C), indicating that *CYLD* downregulation is a frequent consequence of this chromosomal alteration. To determine whether *CYLD* loss contributes to genomic instability, we evaluated genome‐wide instability and homologous recombination deficiency (HRD). *CYLD* deletion was associated with significantly increased genomic instability, as measured by the weighted genomic integrity index (wGII), and elevated HRD scores in both cancer types (Figure 1C,D; Figure S1D,E). Consistent with these findings, functional assays in cell models demonstrated that *CYLD* is required for maintaining genomic integrity. *CYLD*‐deficient PC‐3 cells exhibited increased chromosomal breaks following ionizing radiation (IR) (Figure 1E,F). Alkaline comet assays further showed that *CYLD* depletion exacerbated IR‐induced DNA damage in both PC‐3 and UM‐UC‐3 cells (Figure 1G,H; Figure S1F, G). Analysis of DNA damage response revealed that *CYLD*‐deficient cells displayed elevated γ‐H2AX levels and prolonged persistence following IR, as assessed by immunocytochemistry (ICC) and western blotting (WB) (Figure 1I–L; Figure S1H–K), indicating impaired DNA repair kinetics. Finally, immunohistochemical analysis of prostate (*n* = 28) and bladder (*n* = 27) cancer specimens revealed an inverse correlation between CYLD and γ‐H2AX expression (Figure 1M,N; Figure S1L,M), supporting the in vivo relevance of these findings.

Collectively, these results demonstrate that chromosome 16q loss leads to *CYLD* downregulation, which in turn promotes genomic instability and defective DNA repair.

### 2.2 CYLD Interacts with and Stabilizes TIRR

**FIGURE 2:**
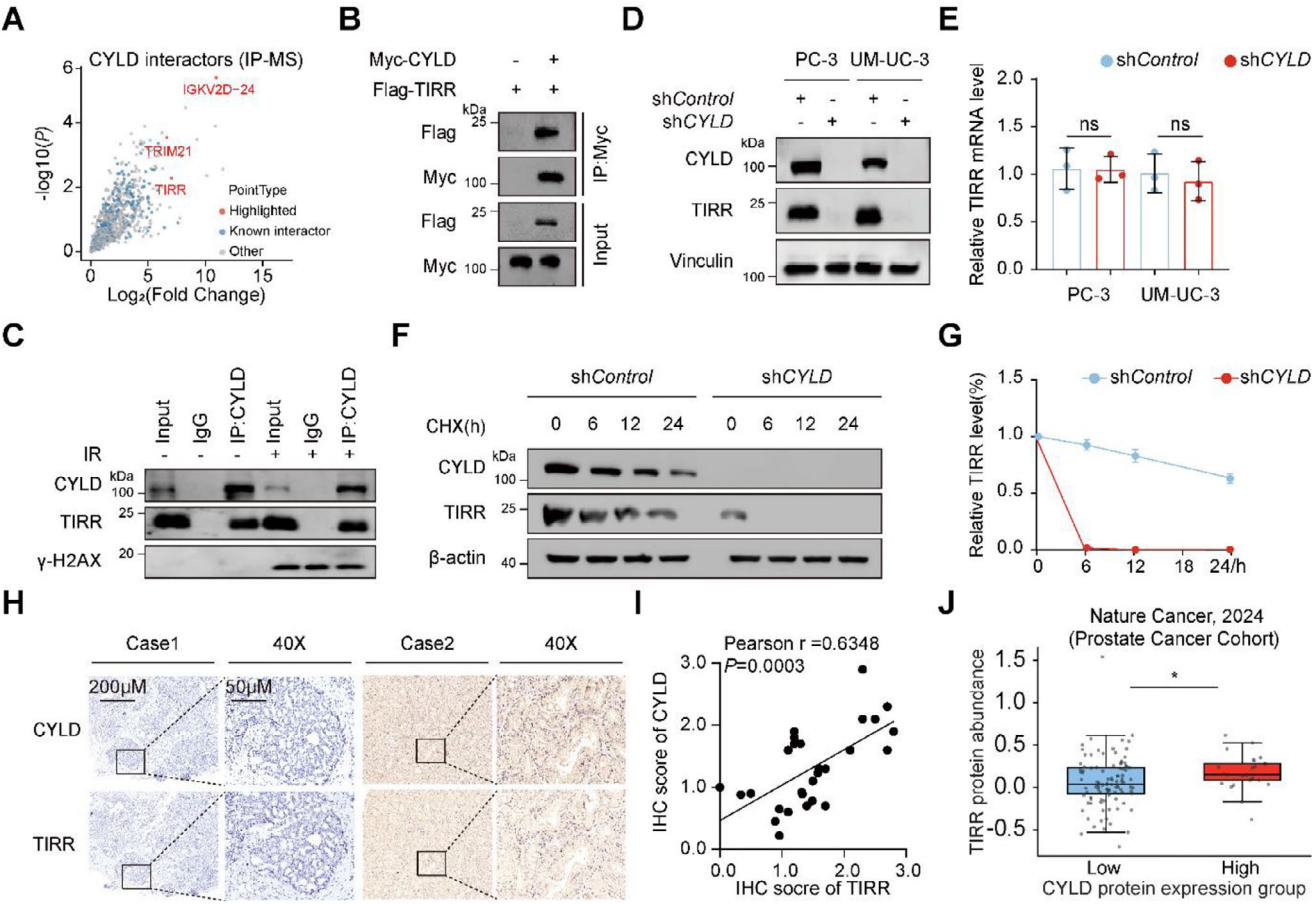
CYLD interacts with and stabilizes TIRR. (A) Co‐IP of whole‐cell lysates from 293T cells expressing HA‐CYLD exposed to IR (6 Gy), analyzed by mass spectrometry. CYLD‐associated proteins are plotted as a volcano plot based on log_2_ Fold changes and –log_10_ PEP scores, with significantly enriched interactors highlighted. (B) Co‐IP of whole‐cell lysates from 293T cells transfected with Myc‐CYLD and Flag‐TIRR. (C) Co‐IP of endogenous TIRR and CYLD in whole‐cell lysates of PC‐3 cells exposed to ionizing radiation. (D)WB analysis of CYLD and TIRR in whole‐cell lysates of control or CYLD knockdown PC‐3 and UM‐UC‐3 cells. (E) mRNA expression of TIRR in control or CYLD knockdown PC‐3 and UM‐UC‐3 cells. Data were shown as the mean ± SD of three independent experiments (*n* = 3). Two‐tailed unpaired Student's *t*‐test; ns, non‐significant. (F and G) WB analysis of TIRR in whole‐cell lysates at indicated time points of CHX treatment (F) and quantification (G) in control and CYLD knockdown PC‐3 cells. Data were shown as the mean ± SD from three biological replicates (*n* = 3). (H and I) Representative images of IHC staining of the indicated antibodies on (H) prostate cancer patient specimens (*n* = 28). Scale bar in 10 X fields: 200 µm. Scale bar in 40 X fields: 50 µm. Correlation analysis between CYLD and γ‐H2AX IHC score was shown in Pearson r = 0.6348; *p* = 0.0003. (J) Differences in TIRR protein abundance in a prostate cancer cohort (CYLD‐Low, *n* = 32; CYLD‐High, *n* = 32) with low or high CYLD protein expression. Two‐tailed unpaired Student's *t*‐test; ^*^ *P*<0.05.

To investigate the molecular mechanisms by which CYLD regulates the DNA damage response, we performed Immunoprecipitation‐mass spectrometry (IP‐MS) to identify CYLD‐interacting proteins. Among the top candidates, several previously reported CYLD‐associated proteins—including DDX3X, TRIM21, and IGKV2D‐24 [2, 28] —were detected, supporting the reliability of the dataset (Figure 2A). Notably, NUDT16L1 (TIRR), a key regulator of 53BP1‐dependent DNA repair [21, 22], was identified as a novel interactor. Co‐immunoprecipitation assays confirmed a specific interaction between CYLD and TIRR in both overexpression and endogenous settings (Figure 2B,C; Figure S2A). Moreover, we observed no significant change in the binding affinity between CYLD and TIRR following DNA damage (Figure 2C; Figure S2A). Functional analysis revealed that CYLD knockdown markedly reduced TIRR protein levels without affecting its mRNA expression (Figure 2D,E), indicating post‐transcriptional regulation. Consistent with this notion, cycloheximide (CHX) chase assays demonstrated that CYLD loss significantly shortened the half‐life of endogenous TIRR protein in both PC‐3 and UM‐UC‐3 cells (Figure 2F,G; Figure S2B,C), supporting a role for CYLD in maintaining TIRR protein stability. Finally, immunohistochemical (IHC) analysis of tumor specimens from prostate (*n* = 28) and bladder (*n* = 25) cancer patients revealed a positive correlation between CYLD and TIRR protein expression (Figure 2H,I; Figure S2D,E). This relationship was further validated in a recently published prostate cancer cohort [29] (Figure 2J; Figure S2F). Together, these findings identify TIRR as a novel CYLD‐interacting protein and establish CYLD as a key regulator of TIRR stability.

### 2.3 CYLD Stabilizes TIRR via K48‐Linked Deubiquitination

**FIGURE 3:**
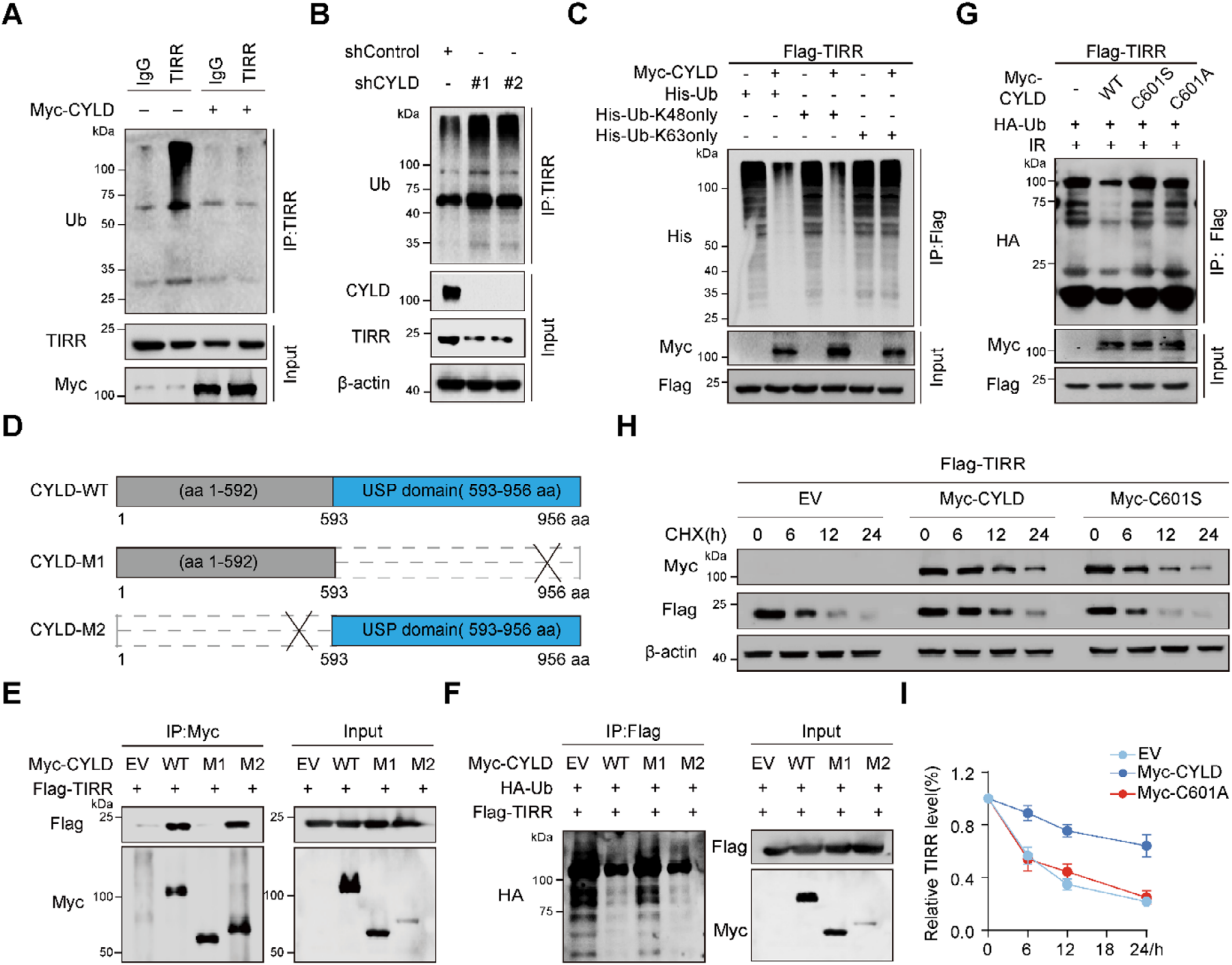
CYLD stabilizes TIRR via K48‐linked deubiquitination. (A) Ubiquitination of endogenous TIRR in PC‐3 cells with ectopic expression of Myc‐CYLD. (B) Immunoblot analysis of ubiquitination of endogenous TIRR in control and CYLD knockdown PC‐3 cells. (C) HEK293T cells were transfected with Flag‐TIRR, His‐Ub (K48‐only or K63‐only), and Myc‐CYLD as indicated, followed by anti‐Flag immunoprecipitation and immunoblotting. (D) Schematic representation of full‐length CYLD and its truncation mutants (M1 and M2), indicating the presence or absence of the USP domain and amino acid boundaries. (E) Interaction analysis between Flag‐TIRR and full‐length or truncated Myc‐CYLD constructs in HEK293T cells by immunoprecipitation and immunoblotting. (F) Ubiquitination analysis of Flag‐TIRR in HEK293T cells expressing His‐Ub and full‐length or truncated Myc‐CYLD constructs following anti‐Flag immunoprecipitation. (G) Ubiquitination of immunoprecipitated Flag‐TIRR in HEK293T cells expressing Myc‐CYLD‐WT or catalytically inactive mutants (C601S or C601A). (H and I) HEK293T cells transfected with Flag‐TIRR and empty vector (EV), Myc‐CYLD‐WT, or Myc‐CYLD‐C601S were treated with CHX (50 µg/µl)for the indicated times and analyzed by immunoblotting (H); quantification is shown in (I).

Given that CYLD functions as a deubiquitinase [30, 31, 32], we next investigated whether it regulates TIRR stability through ubiquitin‐dependent mechanisms. Overexpression of CYLD in PC‐3 cells markedly reduced TIRR polyubiquitination (Figure 3A,B), identifying TIRR as a substrate of CYLD. Ubiquitination assays using linkage‐specific ubiquitin mutants revealed that TIRR is predominantly modified by K48‐linked polyubiquitin chains, which are efficiently removed by CYLD (Figure 3C), indicating that CYLD prevents proteasomal degradation of TIRR. Domain‐mapping studies showed that the interaction between CYLD and TIRR is mediated by the M2 domain of CYLD (Figure 3D,E), which is critical for its deubiquitinase function [33]. GST pull‐down assay using purified recombinant GST‐tagged TIRR and Myc‐tagged CYLD proteins further confirmed this direct binding between CYLD and TIRR in a cell‐free system (Figure S3A). Deletion of this domain abolished CYLD‐mediated suppression of TIRR ubiquitination (Figure 3F). To determine whether CYLD‐mediated deubiquitination of TIRR depends on its catalytic activity, we compared wild‐type (WT) CYLD with catalytically inactive mutants (C601A and C601S) [30, 34, 35]. Re‐expression of WT CYLD, but not the inactive mutants, effectively reduced TIRR ubiquitination (Figure 3G), demonstrating that this process requires CYLD enzymatic activity. Finally, cycloheximide (CHX) chase assays showed that re‐expression of WT CYLD, but not the catalytic mutants, significantly prolonged the half‐life of TIRR protein (Figure 3H,I; Figure S3B,C), confirming that CYLD stabilizes TIRR in a deubiquitinase‐dependent manner. Since we previously reported that the E3 ligase DTX3L regulates TIRR ubiquitination at lysine 187 (K187) on TIRR [24], we further confirmed that while overexpression of CYLD reduced TIRR ubiquitination, it showed less effect on the K187R mutation of TIRR (Figure S3D). Moreover, we observed no direct binding between CYLD and DTX3L. Furthermore, CYLD depletion had no influence on the interaction between DTX3L and TIRR (Figure S3E–G). These results demonstrate that CYLD does not directly modulate DTX3L activity or its binding to TIRR, suggesting that CYLD and DTX3L act independently via parallel mechanisms to regulate TIRR protein dynamics. Collectively, these findings demonstrate that CYLD stabilizes TIRR by selectively removing K48‐linked polyubiquitin chains, thereby preventing its proteasomal degradation.

### 2.4 CYLD Restrains 53BP1 Accumulation at DNA Damage Sites via TIRR

**FIGURE 4:**
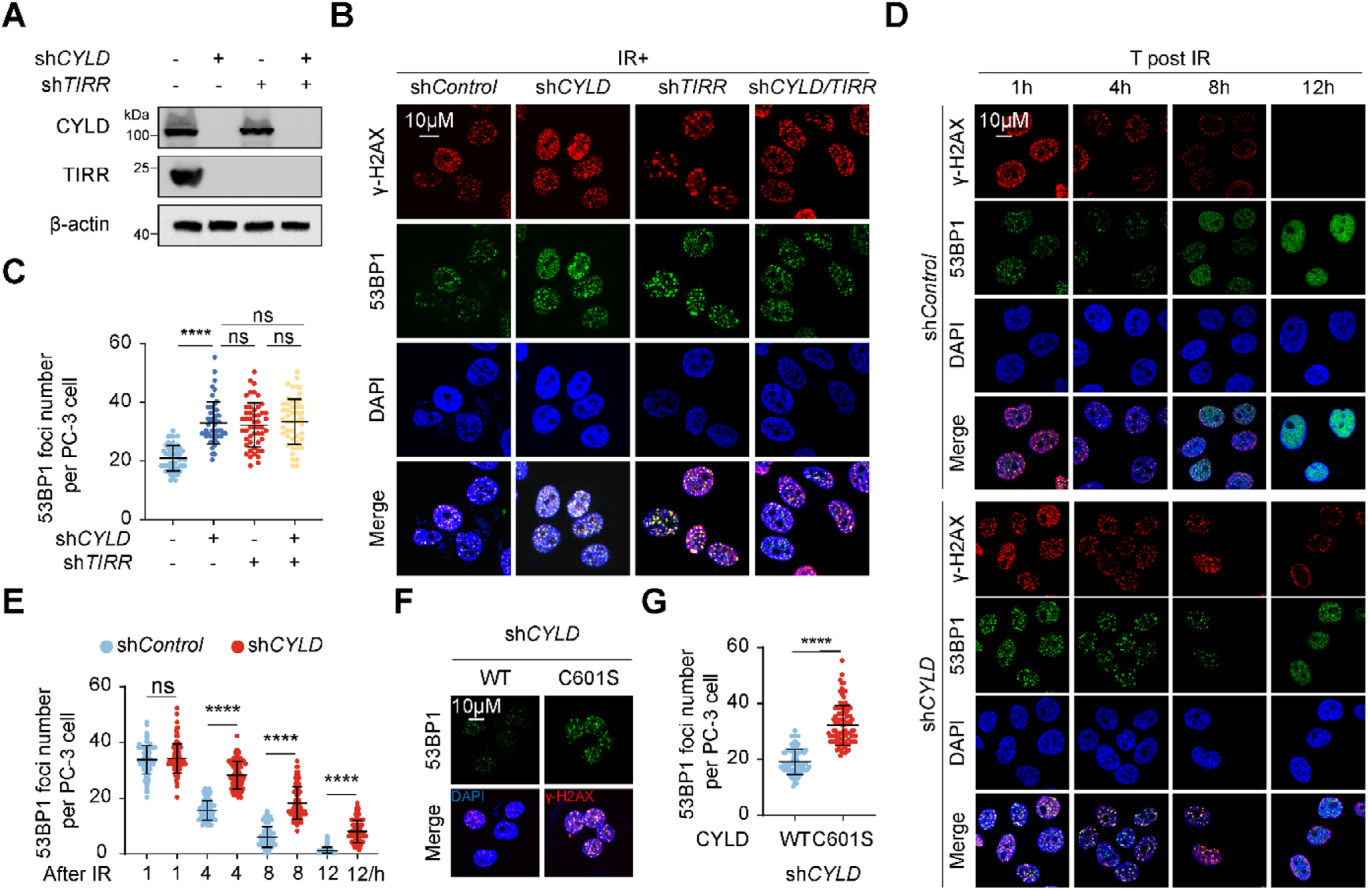
CYLD restrains 53BP1 accumulation at DNA damage sites via TIRR. (A) Immunoblot analysis of CYLD and TIRR in whole‐cell lysates from PC‐3 cells stably expressing control, CYLD‐targeting, TIRR‐targeting, or combined knockdown constructs. (B and C) Representative immunofluorescence images (B) and quantification of 53BP1 (C) foci in PC‐3 cells stably expressing control, CYLD, TIRR, or combined knockdown constructs, with IR (6 Gy). Scale bar, 10 µm. Data are presented as mean ± SD from 20 fields (>50 cells per condition) across three biological replicates. One‐way ANOVA followed by Tukey's multiple‐comparisons test; ns, non‐significant; ^****^ *p* < 0.0001. (D and E) Representative immunofluorescence images (D) and quantification (E) of 53BP1 foci in control or CYLD knockdown PC‐3 cells following IR (6 Gy). Scale bar, 10 µm. Data are presented as mean ± SD from 20 fields (>50 cells per condition) across three biological replicates. One‐way ANOVA followed by Tukey's multiple‐comparisons test; ns, non‐significant; ^****^ *p* < 0.0001. (F and G) Representative immunofluorescence images (F) and quantification of 53BP1 (G) foci in CYLD knockdown PC‐3 cells stably reconstituted with CYLD‐WT or CYLD‐C601S following IR (6 Gy). Scale bar, 10 µm. Data are presented as mean ± SD from 20 fields (>50 cells per condition) across three biological replicates. Two‐tailed unpaired Student's *t*‐test; ^****^ *p* < 0.0001.

Given the established role of TIRR in regulating 53BP1, we next investigated whether CYLD‐mediated stabilization of TIRR influences 53BP1 dynamics following DNA damage [21, 22]. PC‐3 and UM‐UC‐3 cell lines with stable knockdown of CYLD, TIRR, or both were generated, and knockdown efficiency was confirmed by western blotting (Figure 4A; Figure S4A). We observed that depletion of CYLD led to a marked increase in 53BP1 accumulation at DNA double‐strand breaks (DSBs), a phenotype that was recapitulated by TIRR knockdown. Notably, while simultaneous depletion of CYLD and TIRR did not further enhance 53BP1 accumulation, restoration of TIRR expression substantially reduced the hyper‐accumulation of 53BP1 foci induced by CYLD depletion, suggesting that CYLD and TIRR function within the same pathway to regulate 53BP1 recruitment (Figure 4B,C; Figure S4B–I). To further characterize the effect of CYLD on 53BP1 dynamics, we monitored 53BP1 foci formation at 1, 4, 8, and 12 h following ionizing radiation (IR) using immunocytochemistry (ICC). Quantitative analysis revealed that CYLD knockdown significantly prolonged the persistence of 53BP1 foci compared with control cells, indicating sustained retention of 53BP1 at DNA damage sites (Figure 4D,E; Figure S4J,K). Importantly, re‐expression of wild‐type CYLD, but not the catalytically inactive C601S mutant, suppressed the elevated 53BP1 levels observed upon CYLD depletion (Figure 4F,G; Figure S4L,M), demonstrating that this regulatory effect depends on CYLD deubiquitinase activity. Collectively, these findings indicate that CYLD limits 53BP1 accumulation at DNA damage sites, likely through stabilization of TIRR, thereby contributing to proper regulation of the DNA damage response.

### 2.5 CYLD Loss Inhibits the Progression of Homologous Recombination Repair

**FIGURE 5:**
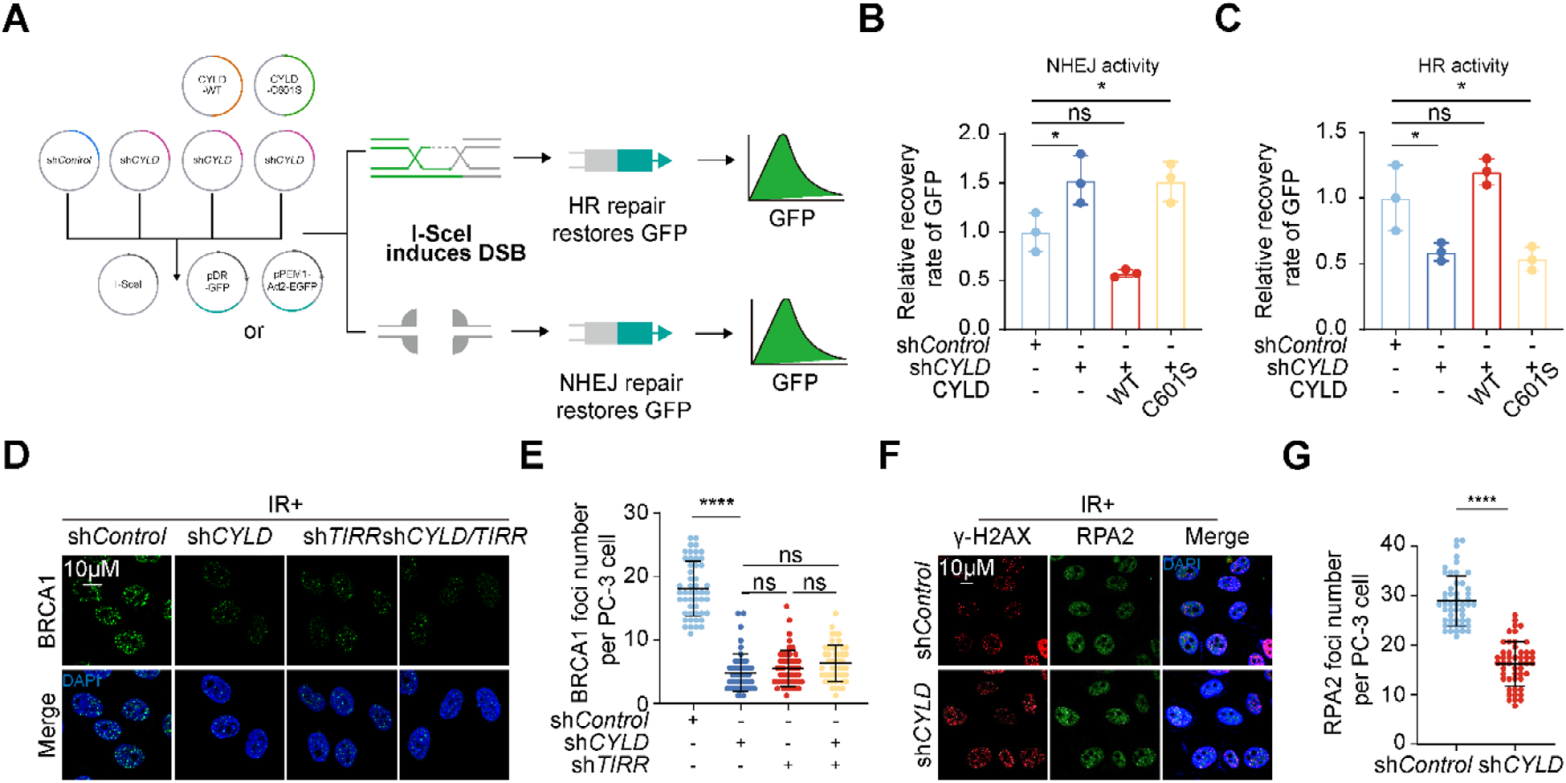
CYLD loss inhibits the progression of homologous recombination repair. (A)Schematic representation of HR and NHEJ reporter assays. (B and C) Analysis of NHEJ (B) and HR (C) activities in control or CYLD knockdown PC‐3 cells transfected with HR or NHEJ reporter constructs in combination with Myc‐CYLD‐WT or Myc‐CYLD‐C601S. Data are presented as mean ± SD from three independent experiments (*n* = 3). One‐way ANOVA followed by Tukey's multiple‐comparisons test; exact *p* values are indicated. (D and E) Representative immunofluorescence images (D) and quantification of BRCA1 (E) foci in PC‐3 cells stably expressing control, CYLD, TIRR, or combined knockdown constructs exposed to IR (6 Gy). Scale bar, 10 µm. Data are presented as mean ± SD from 20 fields (>50 cells per condition) across three biological replicates. One‐way ANOVA followed by Tukey's multiple‐comparisons test; ns, non‐significant; ^****^ *p* < 0.0001. (F and G) Representative immunofluorescence images (F) and quantification of RPA2 (G) foci in control or CYLD knockdown PC‐3 cells following IR (6 Gy). Scale bar, 10 µm. Two‐tailed unpaired Student's *t*‐test; ^****^ *p* < 0.0001.

Because 53BP1 plays a central role in determining DNA repair pathway choice [16, 18, 19], we next assessed the impact of CYLD loss on homologous recombination (HR) and non‐homologous end joining (NHEJ) using reporter assays. CYLD depletion shifted DNA repair toward NHEJ at the expense of HR, and this defect was rescued by re‐expression of wild‐type CYLD, but not by the catalytically inactive C601S mutant (Figure 5A–C), indicating that CYLD enzymatic activity is required for proper HR regulation. Consistently, BRCA1 and RAD51 foci formation following DNA damage was significantly reduced in cells depleted of either CYLD or TIRR. Combined depletion did not further exacerbate this defect, suggesting that CYLD and TIRR function within the same pathway to regulate BRCA1 and RAD51 recruitment to DNA double‐strand breaks (DSBs) (Figure 5D,E; Figure S5A–F). This impairment in HR was further supported by analysis of RPA2, a key factor involved in DNA end resection [36, 37]. Ionizing radiation (IR)‐induced RPA2 foci were markedly decreased in CYLD‐depleted cells compared with controls (Figure 5F,G; Figure S5G,H), indicating defective DNA end processing required for HR.

Together, these findings demonstrate that CYLD loss disrupts homologous recombination by destabilizing TIRR and limiting BRCA1 and RPA2 recruitment to DNA damage sites.

### 2.6 CYLD Loss Enables Synthetic Lethality to PARP Inhibitor in Cancer Cells

**FIGURE 6:**
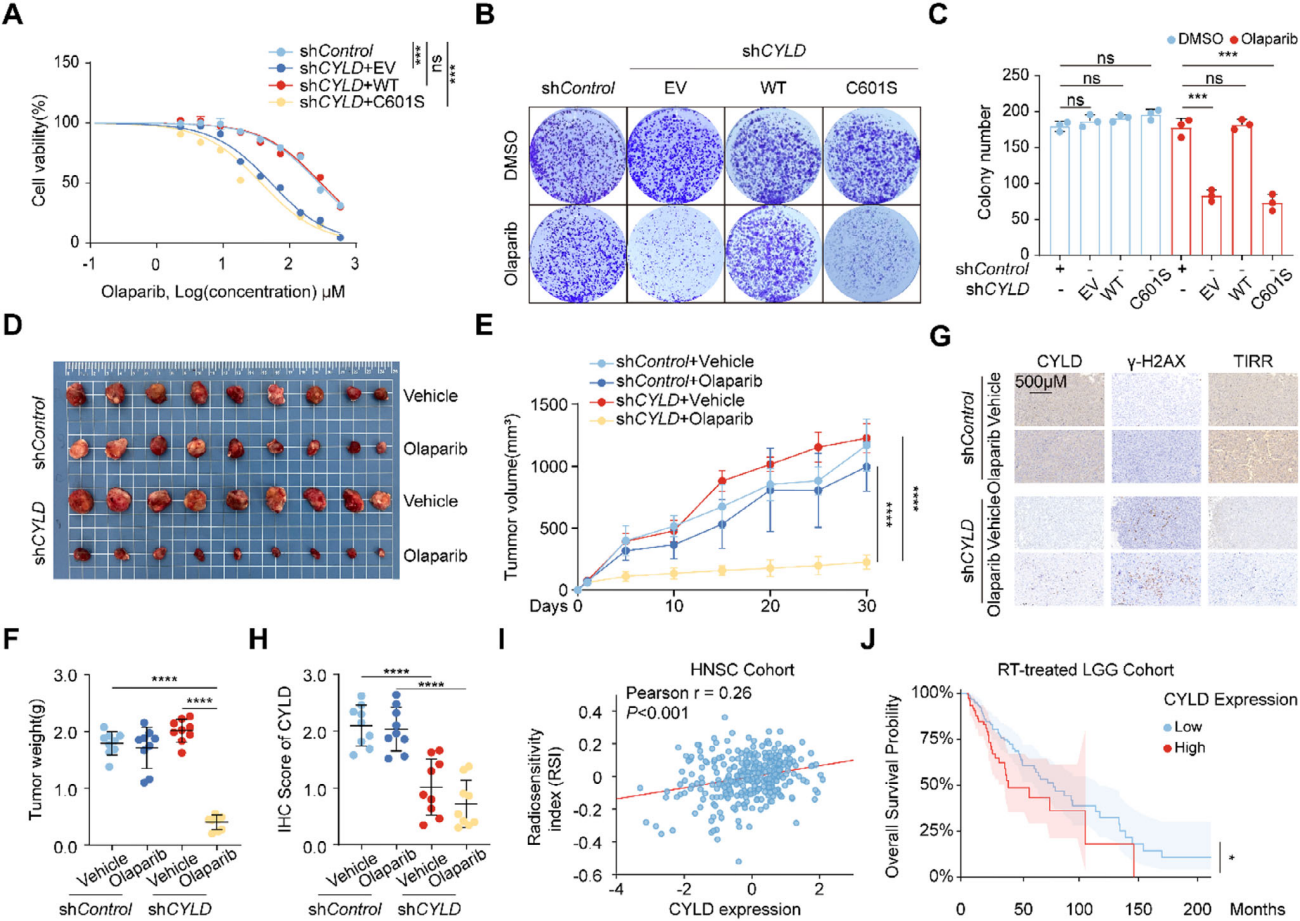
CYLD loss enables synthetic lethality to PARP inhibitor in cancer cells. (A)Dose–response survival curves of control or CYLD‐knockdown PC‐3 cells reconstituted with EV, CYLD‐WT, or CYLD‐C601S and treated with increasing concentrations of Olaparib. Means and SDs are represented. One‐way ANOVA with Tukey's correction for multiple comparisons; *n* = 3. ns, non‐significant; ^***^ *p* < 0.001. (B and C) Colony formation assays of CYLD‐knockdown PC‐3 cells reconstituted with EV, CYLD‐WT, or CYLD‐C601S. Representative images (B) and quantification (C). Data were presented as the mean ± SD of three independent experiments. Two‐way ANOVA followed by Tukey's multiple‐comparisons test; *n* = 3. ns, non‐significant; ^***^ *p* < 0.001. (D‐E) Control or CYLD knockdown PC‐3 cells were injected subcutaneously into SCID mice and treated with vehicle or Olaparib (50 mg/kg). Representative photographed shown in (D). Tumor volume (E). Tumor weight (F). Data in (E and F) are shown as mean ± SD. One‐way ANOVA followed by Tukey's multiple‐comparisons test. (*n* = 9 mice per group). ^****^ *p* < 0.0001. (G‐H) Representative IHC staining of CYLD, γ‐H2AX, and TIRR in tumor tissues from the indicated groups. Scale bar in 40 X fields: 500 µm. Quantification of CYLD IHC scores (H). Two‐way ANOVA followed by Tukey's multiple‐comparisons test; *n* = 9 mice per group; ^****^ *p* < 0.0001. (I) Correlation analysis between radiosensitivity index (RSI) and CYLD expression from the TCGA Head and Neck Squamous Cell Carcinoma (HNSC) Cohort. Pearson r = 0.26; ^***^ *p* < 0.001. (J) Kaplan‐Meier overall survival curves with CYLD low and high expression from the radiotherapy (RT)‐treated LGG cohort. The *p‐*value was calculated using the two‐sided log‐rank test. ^*^ *p* < 0.05.

The frequent loss of CYLD in prostate and bladder cancers, together with our finding that CYLD depletion impairs homologous recombination (HR), prompted us to investigate whether CYLD deficiency renders cancer cells susceptible to PARP inhibitor‐mediated synthetic lethality. Dose–response analyses in PC‐3 and UM‐UC‐3 cells demonstrated that CYLD depletion significantly reduced the IC_50_ of the PARP inhibitor Olaparib compared with control cells (Figure 6A; Figure S6A), indicating enhanced drug sensitivity. Consistently, colony formation assays revealed that Olaparib markedly suppressed clonogenic survival in CYLD‐depleted cells, resulting in fewer and smaller colonies, whereas control cells remained relatively resistant (Figure 6B,C; Figure S6B,C). Importantly, re‐expression of wild‐type CYLD, but not the catalytically inactive C601S mutant, significantly reversed the increased sensitivity to Olaparib (Figure 6A–C; Figure S6A–C). Consistently, TIRR reconstitution also attenuated the increased sensitivity of CYLD‐deficient cells to Olaparib treatment (Figure S6D–I). This finding indicates that CYLD‐mediated resistance to PARP inhibition depends on its deubiquitinase activity and its role in maintaining TIRR stability. To evaluate the therapeutic relevance in vivo, we employed a PC‐3 xenograft model. Olaparib treatment significantly inhibited the growth of CYLD‐deficient tumors compared with control tumors without influencing the mouse body weight (Figure 6D–F; Figure S6J), demonstrating that CYLD loss enhances sensitivity to PARP inhibition in vivo. Immunohistochemical analysis of xenograft tumors further supported our mechanistic findings, revealing an inverse correlation between CYLD and γ‐H2AX expression, and a positive correlation between CYLD and TIRR levels (Figure 6G,H; Figure S6K,L), consistent with observations in cell‐based assays and patient samples. Because public clinical trial datasets tracking PARP inhibitor responses alongside omics data are exceptionally limited, we instead evaluated the clinical relevance of CYLD in the context of radiotherapy—another major DNA double‐strand break‐inducing modality. Using TCGA datasets, we found that CYLD expression is positively associated with the radiation sensitivity index [38]. Furthermore, CYLD loss predicted significantly better overall survival in low‐grade glioma (LGG) patients who received radiotherapy (Figure 6I,J).

Collectively, these results demonstrate that CYLD loss induces an HR‐deficient state that confers vulnerability to PARP inhibitor‐mediated synthetic lethality, highlighting CYLD as a potential predictive biomarker for PARP inhibitor responsiveness in prostate and bladder cancers.

## 3 Conclusion

Loss of chromosome 16q is a recurrent genomic event across multiple cancer types, including breast, bladder, and prostate cancers, and is consistently associated with poor clinical outcomes [39, 40]. Genomic instability commonly arises from defects in chromosome segregation and structural abnormalities, including deletions, translocations, numerical aberrations, amplifications, and trisomy [41, 42, 43]. However, the mechanistic link between chromosome 16q deletion and defective genome maintenance has remained largely unexplored [3]. In this study, we identify CYLD as a major functional contributor to the genomic instability associated with chromosome 16q loss and demonstrate that reduced CYLD expression is closely linked to defective DNA repair and genome maintenance induced by this chromosomal event. (Figure 1). Mechanistically, CYLD deficiency compromises DNA damage repair fidelity, thereby promoting chromosomal instability and tumor progression (Figures 4 and 5). These findings establish a direct molecular connection between chromosome 16q loss and genomic instability. Although our mechanistic studies were primarily performed in prostate and bladder cancer models, the recurrent nature of chromosome 16q deletion across diverse malignancies, together with our validation experiments in chromosome 16q‐deficient MCF‐7 breast cancer cells, suggests that CYLD‐dependent regulation of genome stability may extend beyond these tumor contexts. Nevertheless, because chromosome 16q harbors multiple candidate tumor suppressor loci, whether additional genes cooperate with CYLD loss to drive tumorigenesis remains an important question for future investigation.

CYLD deubiquitinase primarily removes lysine 63 (K63)‐ and lysine 48 (K48)‐linked polyubiquitin chains from its substrates, thereby regulating protein–protein interactions and subcellular localization [32, 33]. Through this molecular function, CYLD serves as a key modulator of multiple signaling pathways, including nuclear factor‐κB, Wnt/β‐catenin, and transforming growth factor‐β signaling [44, 45, 46]. Although emerging evidence has implicated CYLD in the DNA damage response, its precise role in DNA double‐strand break repair pathway choice has remained incompletely defined. Here, we identify TIRR as a previously unrecognized substrate of CYLD and demonstrate that CYLD directly interacts with and stabilizes TIRR through removal of K48‐linked polyubiquitin chains (Figures 2 and 3). Given that TIRR is a critical regulator of the 53BP1 Tudor domain and restricts its chromatin engagement under basal conditions, our findings establish a CYLD–TIRR–53BP1 regulatory axis that governs DNA repair pathway choice (Figure 4). Through this mechanism, CYLD dynamically restrains excessive 53BP1 accumulation at DNA damage sites and preserves homologous recombination repair. Nevertheless, whether CYLD coordinates additional DNA repair substrates or cooperates with other ubiquitin regulators during the DNA damage response remains to be further investigated.

*BRCA1/2* deficiency is the most established cause of homologous recombination deficiency and the principal biomarker guiding PARP inhibitor therapy [47, 48]. Mechanistically, loss of BRCA1/2 impairs homologous recombination repair, rendering tumor cells selectively vulnerable to PARP inhibition through synthetic lethality [49, 50, 51]. However, despite their efficacy in *BRCA*‐mutant tumors, the clinical utility of PARP inhibitors remains limited by a narrow biomarker spectrum and frequent resistance. In the present study, we show that CYLD loss destabilizes TIRR, promotes excessive 53BP1 accumulation at DNA damage sites, and suppresses homologous recombination repair, thereby creating an HR‐deficient state that sensitizes tumor cells to PARP inhibition (Figures 4, 5, 6). These findings identify CYLD loss as a potential HRD biomarker beyond *BRCA1/2* and provide a rationale for expanding PARP inhibitor‐based therapies. Nevertheless, further clinical validation will be required to define its predictive value across tumor types.

In summary, our study defines the CYLD–TIRR–53BP1 axis as a mechanistic link between chromosome 16q loss and defective homologous recombination repair. These findings provide a molecular basis for how 16q deletion drives genomic instability and establishes a therapeutically actionable HR‐deficient state. More broadly, CYLD loss may serve as a biomarker to extend PARP inhibitor‐based strategies beyond the current *BRCA1/2*‐defined setting.

## 4 Experimental Section/Methods

### 4.1 Cell Culture

PC‐3 and UM‐UC‐3 cells were selected as representative models of prostate and bladder cancers, respectively, because both cell lines express detectable endogenous CYLD and are readily amenable to genetic manipulation. PC‐3, UM‐UC‐3, and HEK293T cells were obtained from the American Type Culture Collection (ATCC; Manassas, VA). HEK293T and UM‐UC‐3 cells were maintained in Dulbecco's modified Eagle's medium (DMEM; Gibco, 11965092) supplemented with 10% fetal bovine serum (FBS; Thermo Fisher Scientific, 30067334). PC‐3 cells were cultured in RPMI‐1640 medium (Gibco, 21870092) supplemented with 10% FBS. All cells were grown at 37°C in a humidified incubator with 5% CO_2_. Cells were routinely tested for mycoplasma contamination and confirmed to be negative.

### 4.2 Antibodies and Chemicals

Primary antibodies used in this study included anti‐NUDT16L1 (Sigma‐Aldrich, HPA044186; 1:1000), anti‐53BP1 (Abcam, ab36823; 1:1000), anti‐CYLD (Proteintech, 66858‐1‐Ig; 1:1000), anti‐Vinculin (Santa Cruz Biotechnology, sc‐73614; 1:1000), anti‐Myc (Santa Cruz Biotechnology, sc‐40; 1:1000), anti‐FLAG (Cell Signaling Technology, 8146; 1:1000), anti‐HA (Cell Signaling Technology, 3724; 1:1000), anti‐ubiquitin (Cell Signaling Technology, 14049; 1:1000), and anti–phospho‐histone H2A.X (Ser139) (Cell Signaling Technology, 9718 or 80312S; 1:1000).Secondary antibodies included rabbit IgG (H+L)–FITC (Xi'an Zhuangzhi Biotechnology Co., Ltd., EK023; 1:500), rabbit IgG (H+L)–Cy3 (Xi'an Zhuangzhi Biotechnology Co., Ltd., EK022; 1:500), rabbit IgG (H+L)–Alexa Fluor (Abcam, ab150079; 1:500), mouse IgG (H+L)–FITC (Xi'an Zhuangzhi Biotechnology Co., Ltd., EK013; 1:500), mouse IgG (H+L)–Cy3 (Xi'an Zhuangzhi Biotechnology Co., Ltd., EK012; 1:500), HRP‐conjugated rabbit IgG (Abclonal, AS014; 1:5000), and HRP‐conjugated mouse IgG (Abclonal, AS003; 1:5000). The following chemicals were used: Olaparib (Selleck, #S1060), Cycloheximide (MCE, #HY‐12320) and MG132 (Sigma‐Aldrich, #133407‐82‐6).

### 4.3 Transfection and Virus Infection

Transient transfections were performed using polyethylenimine (PEI; Polysciences, 23966‐2) or Lipofectamine 2000 (Thermo Fisher Scientific, 11668019) according to the manufacturers’ instructions. Lentiviral packaging plasmids together with pLKO.1‐based shRNA constructs targeting CYLD or TIRR, or CYLD and TIRR expression constructs, were transfected into HEK293T cells. Viral supernatants were collected at 48 h and 72 h post‐transfection. Target cancer cells were infected with virus‐containing supernatants in the presence of polybrene (8 µg/ml) and subsequently selected in complete medium containing puromycin (1.5 µg/ml).

### 4.4 RNA Interference and shRNA‐Mediated Gene Deletion

The sequences for CYLD shRNA were 5′‐GGCGCGAGCTGCTCTTCAACGCTCGAGCGTTGAAGAGCAGCTCGCGCC‐3′ (#1), 5′‐AGGATGTTGTAGAGATAAATGCTCGAGCATTTATCTCTACAACATCCT‐3′ (#2) and 5′‐GATTGTTACTTCTATCAAATTCTCGAGAATTTGATAGAAGTAACAATC‐3′ (#3). The sequence for TIRR shRNAs were 5′‐GTGCTGATGCAGATGCGTTTCCTCGAGGAAACGCATCTGCATCAGCACT‐3′ (#1),

5′‐CTAAGTGCCAGCTCCTCTTTGCTCGAGCAAAGAGGAGCTGGCACTTAGT‐3′ (#2) and 5′‐CAACTTCCTGAGCAACGCCTTCTCGAGAAGGCGTTGCTCAGGAAGTTGT‐3′ (#3).

### 4.5 CRISPR–Cas9‐Mediated CYLD Knockout

CRISPR‐Cas9‐mediated knockout of CYLD was generated using lentiviral transduction. Single‐guide RNAs (sgRNAs) targeting human CYLD were designed and cloned into the lentiCRISPR v2 vector (Addgene #52961), which co‐expresses Cas9 and puromycin resistance genes. The sgRNA sequences used in this study were as follows:

sgCYLD#1: 5′‐ CACCGTCACTGACGGGGTGTACCAA‐3′

sgCYLD#2: 5′‐ CACCG AATGATGCAAACCTAGAGTC‐3′

sgCYLD#3: 5′‐ CACCG TATGGGGTAATCCGTTGGAT‐3′

A non‐targeting sgRNA was used as the control:

sgCtrl: 5′‐ GCGAGGTATTCGGCTCCGCG‐3′. Lentiviral particles were produced in HEK293T cells by co‐transfecting lentiCRISPR v2 constructs with psPAX2 and pMD2.G packaging plasmids using Lipofectamine 3000 (Invitrogen). Viral supernatants were collected at 48 and 72 h after transfection, filtered through a 0.45‐µm filter, and used to infect PC‐3 and UM‐UC‐3 cells in the presence of 8 µg/mL polybrene (Sigma–Aldrich). 24 h after infection, cells were selected with puromycin (2 µg/mL) for 5–7 days. CYLD depletion was confirmed by immunoblot analysis prior to subsequent experiments.

### 4.6 Plasmids and Mutagenesis

The FLAG‐tagged NUDT16L1 expression construct was purchased from MiaolingBio (China; P28448). Myc‐tagged and HA‐tagged CYLD expression plasmids were obtained from MiaolingBio (China; P49831 and P35685, respectively). Site‐directed mutagenesis of Myc‐CYLD was performed using the KOD Plus Mutagenesis Kit (TOYOBO, SMK‐101) according to the manufacturer's instructions. The lentiCRISPR v2 plasmid was obtained from Addgene (52961).

### 4.7 Immunoblotting and Immunoprecipitation

Cells were harvested and lysed in immunoprecipitation (IP) buffer [50 mm Tris‐HCl (pH 7.4), 150 mM NaCl and 0.1% NP‐40] supplemented with protease inhibitors [Complete Mini, Roche, 11836153001] and phosphatase inhibitors (cocktail sets I and II, Calbiochem, 80501‐130). Lysates were briefly sonicated to ensure complete cell disruption and subsequently clarified by centrifugation at 13 000 × g for 10 min at 4°C. Protein concentrations were determined using a bicinchoninic acid (BCA) protein assay. For immunoblotting, protein samples were mixed with 5× SDS sample buffer (250 mM Tris‐HCl, pH 6.8; 10% SDS; 25 mm β‐mercaptoethanol; 30% glycerol; 0.05% bromophenol blue) and boiled for 8 min. Equal amounts of protein were resolved by SDS‐polyacrylamide gel electrophoresis (SDS–PAGE) and transferred to nitrocellulose membranes. Membranes were blocked with 5% non‐fat milk for 1 h at room temperature and incubated with primary antibodies at 4°C overnight, followed by incubation with appropriate secondary antibodies for 1 h at room temperature. The protein bands were visualized by ECL reagents, imaged on a ChemiDoc XRS+ Imager, and analyzed with Image Lab Software. For immunoprecipitation, clarified cell lysates were further centrifuged at 13 000 rpm for 15 min at 4°C and incubated with primary antibody‐conjugated protein A/G beads (Thermo Fisher Scientific, 20423) or HA‐ or FLAG‐conjugated agarose beads (Sigma‐Aldrich, A2095 or A2220) with rotation at 4°C overnight. Beads were washed extensively with IP buffer, and bound proteins were eluted by boiling in SDS sample buffer at 95°C for 5 min, followed by SDS‐PAGE and immunoblotting analysis.

### 4.8 Fluorescence Immunohistochemistry

PC‐3 or UM‐UC‐3 cells were seeded on 13‐mm glass coverslips, fixed with 4% paraformaldehyde for 15 min, and permeabilized with 0.2% Triton X‐100 for 10 min at room temperature. Cells were blocked with 5% bovine serum albumin (BSA) for 1 h and incubated with primary antibodies for 2 h at room temperature, followed by washing at least three times with PBS containing 0.01% Tween‐20 (PBS‐T). Samples were then incubated with fluorophore‐conjugated secondary antibodies for 1 h at room temperature. After three additional washes with PBS‐T, coverslips were mounted onto glass slides using mounting medium containing 4′,6‐diamidino‐2‐phenylindole (DAPI). Images were acquired using a confocal microscope. For quantification of 53BP1 or γ‐H2AX foci, 10 randomly selected fields comprising more than 100 cells were analyzed for each experimental condition. The number of foci per cell and the integrated optical density were quantified using ImageJ software (version 1.53, National Institutes of Health). Approximately 100 cells were analyzed per experiment.

### 4.9 Karyotype Analysis

PC‐3 cells were exposed to ionizing radiation (IR) and treated with colcemid 1 h prior to harvest to arrest cells in metaphase. Cells were washed twice with phosphate‐buffered saline (PBS) and resuspended in hypotonic solution (0.075 M KCl) at 37°C for 45 min. Cells were fixed twice in freshly prepared fixative (methanol: glacial acetic acid, 3:1) for 15 min each. Fixed cell suspensions were dropped onto glass slides and air‐dried, followed by staining with Diff‐Quik stain for 1 min. Approximately 100 metaphase spreads with well‐separated chromosomes were captured and analyzed for each experimental group.

### 4.10 Alkaline Comet Assay

DNA damage was evaluated using an alkaline comet assay kit (Beyotime Biotechnology, China) according to the manufacturer's instructions. PC‐3 and UM‐UC‐3 cells were harvested, washed with ice‐cold phosphate‐buffered saline (PBS), and subjected to comet assay analysis. Briefly, cells were mixed with low‐melting‐point agarose and spread onto comet assay slides, followed by solidification at 4°C. Slides were immersed in lysis buffer at 4°C in the dark, subjected to alkaline DNA unwinding and electrophoresis under alkaline conditions as specified by the kit protocol, and subsequently neutralized. DNA was stained with a fluorescent DNA‐binding dye, and comet images were acquired using a fluorescence microscope. DNA damage was quantified by measuring the olive tail moment using CometScore software. At least 100 cells were analyzed per sample, and experiments were performed at least three times independently.

### 4.11 Mass Spectrometry Analysis

Liquid chromatography–mass spectrometry (LC–MS/MS) analysis was performed essentially as previously described. HEK293T cells were transfected with HA‐tagged CYLD and harvested 48 h post‐transfection. Cells were lysed in immunoprecipitation (IP) buffer, and protein complexes were immunoprecipitated using control IgG agarose beads (Cell Signaling Technology, 37478) or HA‐conjugated agarose beads (Sigma–Aldrich, A2220). Bound proteins were eluted with 2% SDS and digested overnight with sequencing‐grade modified trypsin (Promega, V5111). Peptides were analyzed using a nanoflow EASY‐nLC 1200 system (Thermo Fisher Scientific, Odense, Denmark) coupled to an Orbitrap Exploris 480 mass spectrometer (Thermo Fisher Scientific, Bremen, Germany). Raw data were searched against the UniProt human protein database (75 004 entries; downloaded on July 1, 2020) using Protein Discoverer software (version 2.4.1.15, Thermo Fisher Scientific) and Mascot (version 2.7.0, Matrix Science).

### 4.12 Ubiquitination Assay

HEK293T cells were transfected with HA‐tagged ubiquitin (HA‐Ub), FLAG‐tagged NUDT16L1, and other plasmids as indicated. 24 h after transfection, cells were treated with the proteasome inhibitor MG132 (10 µM) for 6 h and subsequently lysed in immunoprecipitation (IP) buffer on ice for at least 10 min. Lysates were briefly sonicated and clarified by centrifugation at 16 000 × g for 15 min at 4°C. The supernatants were incubated with FLAG‐conjugated agarose beads (Sigma–Aldrich) with rotation at 4°C overnight. Beads were washed extensively with IP buffer, and bound proteins were eluted by boiling in SDS sample buffer at 95°C for 8 min. Eluted proteins were resolved by SDS‐polyacrylamide gel electrophoresis (SDS–PAGE) and analyzed by immunoblotting.

### 4.13 Tissue Microarray Immunohistochemistry (IHC)

Prostate cancer and bladder cancer tissue samples were obtained from the First Affiliated Hospital of Xi'an Jiaotong University (Xi'an, China) with approval from the institutional Ethics Committee and written informed consent from all patients. Formalin‐fixed, paraffin‐embedded tumor tissues were sectioned at 4 µm and subjected to immunohistochemical staining using the indicated antibodies following standard protocols. Images were acquired using an Olympus microscope with digital imaging software. Protein expression was semiquantitatively evaluated based on staining intensity (0–3) and the proportion of positive cells, and an immunoreactivity score was calculated by multiplying these values. All slides were evaluated by the same experienced pathologist in a blinded manner.

### 4.14 Quantitative Real‐Time Polymerase Chain Reaction (Real‐Time qPCR)

Total RNA (0.5–1 µg) was isolated using RNAfast 200 reagents according to the manufacturer's instructions, and RNA concentration was measured at 260 nm using a multiwavelength microplate reader. Reverse transcription was performed using PrimeScript RT Master Mix. Quantitative real‐time PCR was carried out using 2× SYBR Green qPCR Master Mix, and relative mRNA expression levels were normalized to 18S rRNA. Relative gene expression was calculated using the 2^−ΔΔCt method.

### 4.15 HR and NHEJ Reporter Assay

PC‐3 cells were transfected with the indicated plasmids or shRNA constructs, either individually or in combination, together with HR (pDR‐GFP) or NHEJ (pPEM1‐Ad2‐EGFP) reporter constructs and an expression vector encoding the I‐SceI endonuclease. GFP expression induced by a positive control plasmid was used to normalize transfection efficiency. Cells were cultured for 48 h and subsequently analyzed by flow cytometry.

### 4.16 3‐(4,5‐Dimethylthiazol‐2‐yl)‐2,5‐Diphenyltetrazolium Bromide (MTT) Assay

Cells were seeded in 96‐well plates at a density of 3000 cells per well. After 24 h, cells were treated with the indicated concentrations of drugs for 48 h. The culture medium was then replaced with fresh medium containing MTT (0.5 mg ml^−^^1^), and cells were incubated for 2 h at 37°C. The medium was aspirated, DMSO was added to dissolve the formazan crystals, and plates were shaken on an orbital shaker for 10 min. Absorbance was measured at 570 nm. Each condition was assayed in triplicate, background absorbance from medium‐only wells was subtracted, and quantitative analysis was performed.

### 4.17 Colony Formation Assay

For clonogenic survival assays, cells were seeded in six‐well plates at appropriate densities (typically 1000 cells per well) according to the indicated treatments. After 24 h, cells were treated with DMSO or the indicated concentrations of drugs and cultured for 10–14 days to allow colony formation. Colonies were then fixed with 4% paraformaldehyde for 15 min and stained with 0.5% (w/v) crystal violet. After washing with water, colonies containing more than 50 cells were counted and analyzed.

### 4.18 Generation and Treatment of Prostate Cancer Xenografts in Mice

PC‐3 cells (5 × 10^6^) stably expressing sh*Control* or sh*CYLD* were subcutaneously injected into the right flanks of six‐week‐old male SCID mice. When xenograft tumors reached a volume of approximately 100 mm^3^, mice were treated with vehicle or olaparib (50 mg kg^−^^1^, intraperitoneal injection, once daily; Selleck, S1060). Tumor volumes were measured every 3 days for up to 30 days and calculated using the formula: 0.5 × length (L) × width (W)^2^. At the end of the experiment, tumors were excised, photographed, and weighed. All animal experiments were conducted in accordance with the Rules for Animal Experiments of the Chinese Government and were approved by the Ethics Committee of the First Affiliated Hospital of Xi'an Jiaotong University (Xi'an, China).

### 4.19 Quantification and Statistical Analysis

Statistical analyses were performed using GraphPad Prism 8. Comparisons between two groups were conducted using two‐tailed unpaired Student's *t*‐tests. Comparisons among three or more groups were performed using one‐way ANOVA followed by Tukey's multiple‐comparisons test. For experiments involving two independent variables, one‐way ANOVA followed by Tukey's multiple‐comparisons test and two‐way ANOVA followed by Tukey's multiple‐comparisons test were applied. Non‐parametric analyses were performed using two‐sided Wilcoxon tests. Data are presented as mean ± SD unless otherwise indicated. Sample sizes (n) are provided in the corresponding figure legends. Statistical significance is denoted as ns, not significant; ^*^ *p* < 0.05; ^**^ *p* < 0.01; ^***^ *p* < 0.001; and ^****^ *p* < 0.0001. Exact *p*‐values and 95% confidence intervals are reported for survival and correlation analyses whenever applicable.

## Author Contributions

**Qi Ye**: methodology, investigation, validation, data curation. **Mingming Lu**: validation, investigation, data curation, writing – original draft, formal analysis. **Tao Liu**: writing – review and editing, data curation. **Zixi Wang**: software, formal analysis, data curation. **Bin Wang**: funding acquisition. **Lei Li**: software, formal analysis, data curation. **Yuzeshi Lei**: formal analysis, data curation. **Yong Zhang**: project administration, resources, supervision, writing – review and editing. **Tianjie Liu**: conceptualization, visualization. **Ke Wang**: conceptualization, software. **Jialu Kang**: methodology, data curation, investigation, validation, writing – original draft. **Shan Xu**: conceptualization, software, visualization. **Jian Ma**: project administration, writing – review and editing, resources, supervision, writing – original draft. **Leihong Ye**: data curation, resources.

## Conflicts of Interest

The authors declare no conflicts of interest.

## Supporting information




**Supporting File 1**: advs77298‐Sup‐0001‐suppMat.doc.

**Supporting File 2**: advs77298‐sup‐0002‐SuppMat.doc.

## Data Availability

The data that support the findings of this study are available on request from the corresponding author. The data are not publicly available due to privacy or ethical restrictions.
