# Supplementary material for: Loss of CYLD on Chromosome 16q Impairs Homologous Recombination and Genomic Stability Through TIRR Degradation
Source: Adv Sci (Weinh). 2026 Aug 21;13(62):e77298. doi: 10.1002/advs.77298 (PMC13496282; doi:10.1002/advs.77298)
Supplement: Supplementary file 1 [file ADVS-13-e77298-s001.doc]

Supporting Information

Title

Loss of CYLD on chromosome 16q impairs homologous recombination and genomic stability through TIRR degradation

Mingming Lu, Jialu Kang, Qi Ye, Zixi Wang, Lei Li, Yuzeshi Lei, Leihong Ye, Tianjie Liu, Bin Wang, Tao Liu, Shan Xu, Ke Wang, Yong Zhang, Jian Ma, Lei Li

Correspondence: Yong Zhang (yongzhang761@mail.xjtu.edu.cn), Jian Ma (majian0922@gmail.com), Lei Li (lilydr@163.com)

**Figure S1. Chromosome 16q loss drives CYLD downregulation and genomic instability in cancer. Related to Figure 1.**

(A) Immunoblot analysis in MCF-7 cells expressing Flag-CYLD or the indicated chromosome 16q-associated DNA damage repair genes following ionizing radiation (IR) treatment.

(B) Homologous recombination (HR) reporter assay in MCF-7 cells expressing the indicated constructs. Data are presented as mean ± SD from three independent biological replicates (*n* = 3). Statistical significance was determined by one-way ANOVA followed by Tukey's multiple-comparisons test. ns, not significant; ****P* < 0.001.

(C)Analysis of CYLD mRNA expression across chr16q copy-number status in bladder cancer samples from TCGA. CYLD mRNA expression is shown as log2(RSEM + 1). Kruskal-Wallis test followed by Dunn’s multiple-comparisons test; ***P* < 0.01.

(D and E) Analysis of weighted genome integrity index (wGII) score (D) and homologous recombination deficiency (HRD) score (E) in bladder cancer samples. The box outlines show the 25th and 75th percentiles, and the solid lines show the median value. Means and SDs are represented. Kruskal-Wallis test followed by Dunn’s multiple-comparisons test; **P* < 0. 01; ****P* < 0.001.

(F and G) Genomic stability analysis with comet assay in UM-UC-3 cells. Cells were treated with or without IR(2Gy) for 2 hours before being harvested. Scale bar, 50 µm. Analysis of comet tail shown in (E) using the Comet Assay Software Project. Means and SDs are represented. Two-way ANOVA followed by Tukey's multiple-comparisons test; ns, non-significant; *****P* < 0.0001.

(H and I) WB analysis of CYLD and γ-H2AX in whole-cell lysates of control or CYLD knockdown UM-UC-3 cells post IR (6Gy). (I)Quantification of relative CYLD and γ-H2AX expression was performed across three biologically independent experiment.

(J and K) Immunofluorescence analysis (H) and quantification (I) of CYLD and γ-H2AX in control or CYLD knockdown UM-UC-3 cells treated with or without IR (6 Gy). Scale bar, 10 µm. Data were shown as the mean ± SD from 20 fields (>100 cells, *n*=20) from three biological replicates. One-way ANOVA followed by Tukey's multiple-comparisons test; ns, non-significant; ****P* < 0.001.

(L)Representative images of IHC staining of CYLD and γ-H2AX protein from bladder cancer samples (*n* = 27). Scale bar in 10 X fields: 500 µm. Scale bar in 40 X fields: 100 µm.

(M) Correlation analysis between CYLD and γ-H2AX IHC score in tumor tissues. Pearson r = -0.7186; *****P* < 0.0001.

**Figure S2. CYLD interacts with and stabilizes TIRR. Related to Figure 2.**

(A) Co-IP of endogenous TIRR and CYLD in whole-cell lysates of PC-3 cells and exposed to ionizing radiation.

(B and C) WB analysis of TIRR in whole-cell lysates at indicated time points of CHX treatment (B) and quantification (C) in control and CYLD knockdown UM-UC-3 cells. Data were shown as the mean ± SD from three biological replicates (*n* = 3).

(D and E) Representative images of IHC staining of indicated antibodies on (D) bladder cancer patient specimens (*n* = 25). Scale bar in 10 X fields: 200 µm. Scale bar in 40 X fields: 50 µm. Correlation analysis between CYLD and γ-H2AX IHC score was shown in (E) Pearson r = 0.7884; *P* < 0.0001.

(F) Spearman correlation analysis between CYLD-TIRR axis and adjusted γ-H2AX level was shown in (E) Spearman ρ= -0.297; **P* < 0.05.

**Figure S3. CYLD stabilizes TIRR via K48-linked deubiquitination. Related to Figure 3.**

(A) GST pull-down assay showing the direct interaction between TIRR and CYLD. Purified GST or GST-TIRR fusion proteins were incubated with lysates containing Myc-tagged CYLD wild-type (WT) or truncation mutants (M1 and M2). Coomassie blue staining shows the purification of GST and GST-TIRR proteins.

(B and C) HEK293T cells transfected with Flag-TIRR and empty vector (EV), Myc-CYLD-WT, or Myc-CYLD-C601A were treated with CHX (50 μg/μl)for the indicated times and analyzed by immunoblotting (B)；quantification is shown in (C).

(D) In vivo ubiquitination assay examining the effect of CYLD on TIRR ubiquitination. Cells were co-transfected with Flag-TIRR (WT or the ubiquitination-defective mutant K187R), HA-tagged ubiquitin, and Myc-CYLD as indicated. Flag-TIRR was immunoprecipitated and ubiquitinated species were detected using an anti-HA antibody.

(E and F) Endogenous co-immunoprecipitation demonstrating the interaction between DTX3L and CYLD.

(G) Co-immunoprecipitation assay examining the interaction between DTX3L and TIRR in control and CYLD-deficient cells. Cells expressing Myc-DTX3L and Flag-TIRR were subjected to Flag immunoprecipitation followed by immunoblotting with anti-Myc and anti-Flag antibodies.

**Figure S4. CYLD restrains 53BP1 accumulation at DNA damage sites via TIRR. Related to Figure 4.**

(A)Immunoblot analysis of CYLD and TIRR in whole-cell lysates from UM-UC-3 cells stably expressing control, CYLD-targeting, TIRR-targeting, or combined knockdown constructs.

(B and C) Representative immunofluorescence images (B) and quantification of 53BP1 (C) foci in UM-UC-3 cells stably expressing control, CYLD, TIRR, or combined knockdown constructs, with IR (6 Gy). Scale bar, 10 µm. Data are presented as mean ± SD from 20 fields (>50 cells per condition) across three biological replicates. One-way ANOVA followed by Tukey's multiple-comparisons test. *****P* < 0.0001.

(D)Immunoblot analysis of CYLD and TIRR in whole-cell lysates from PC-3 cells stably expressing indicated constructs.

(E and F) Representative immunofluorescence images (E) and quantification of 53BP1 (F) foci in PC-3 cells stably expressing indicated constructs, with IR (6 Gy). Scale bar, 10 µm. Data are presented as mean ± SD from 20 fields (>50 cells per condition) across three biological replicates. One-way ANOVA followed by Tukey's multiple-comparisons test; ns, non-significant; *****P* < 0.0001.

(G)Immunoblot analysis of CYLD and TIRR in whole-cell lysates from UM-UC-3 cells stably expressing indicated constructs.

(H and I) Representative immunofluorescence images (H) and quantification of 53BP1 (I) foci in UM-UC-3 cells stably expressing indicated constructs, with IR (6 Gy). Scale bar, 10 µm. Data are presented as mean ± SD from 20 fields (>50 cells per condition) across three biological replicates. One-way ANOVA followed by Tukey's multiple-comparisons test; ns, non-significant; *****P* < 0.0001.

(J and K) Representative immunofluorescence images (J) and quantification (K) of 53BP1 foci in control or CYLD knockdown UM-UC-3 cells following IR (6 Gy). Scale bar, 10 µm. Data are presented as mean ± SD from 20 fields (>50 cells per condition) across three biological replicates. One-way ANOVA followed by Tukey's multiple-comparisons test; ns, non-significant; *****P* < 0.0001.

(L and M) Representative immunofluorescence images (L) and quantification of 53BP1 (M) foci in CYLD knockdown UM-UC-3 cells stably reconstituted with CYLD-WT or CYLD-C601S following IR (6 Gy). Scale bar, 10 µm. Data are presented as mean ± SD from 20 fields (>50 cells per condition) across three biological replicates. Two-tailed unpaired Student’s t-test;*****P* < 0.0001.

**Figure S5. CYLD loss inhibits the progression of homologous recombination repair. Related to Figure 5.**

(A and B) Representative immunofluorescence images (A) and quantification of BRCA1 (B) foci in UM-UC-3 cells stably expressing control, CYLD, TIRR, or combined knockdown constructs exposed to IR (6 Gy). Scale bar, 10 µm. Data are presented as mean ± SD from 20 fields (>50 cells per condition) across three biological replicates. One-way ANOVA followed by Tukey's multiple-comparisons test; *****P* < 0.0001.

(C and D) Representative immunofluorescence images (C) and quantification of RAD51 (D) foci in PC-3 cells stably expressing control, CYLD, TIRR, or combined knockdown constructs exposed to IR (6 Gy). Scale bar, 10 µm. Data are presented as mean ± SD from 20 fields (>50 cells per condition) across three biological replicates. One-way ANOVA followed by Tukey's multiple-comparisons test; ns, non-significant; *****P* < 0.0001.

(E and F) Representative immunofluorescence images (E) and quantification of RAD51 (F) foci in UM-UC-3 cells stably expressing control, CYLD, TIRR, or combined knockdown constructs exposed to IR (6 Gy). Scale bar, 10 µm. Data are presented as mean ± SD from 20 fields (>50 cells per condition) across three biological replicates. One-way ANOVA followed by Tukey's multiple-comparisons test; ns, non-significant; *****P* < 0.0001.

(G and H) Representative immunofluorescence images (G) and quantification of RPA2 (H) foci in control or CYLD knockdown UM-UC-3 cells following IR (6 Gy). Scale bar, 10 µm. Two-tailed unpaired Student’s t-test; *****P* < 0.0001.

**Figure S6. CYLD knockout enables synthetic lethality to PARP inhibitor in cancer cells. Related to Figure 6.**

(A)Dose–response survival curves of control or CYLD-knockdown UM-UC-3 cells reconstituted with EV,CYLD-WT or CYLD-C601S and treated with increasing concentrations of Olaparib. Means and SDs are represented. One-way ANOVA with Tukey's correction for multiple comparisons; *n*=3. ns, non-significant; ****P* < 0.0001.

(B and C) Colony formation assays of CYLD-knockdown UM-UC-3 cells reconstituted with EV, CYLD-WT or CYLD-C601S. Representative images (B) and quantification (C). Data were presented as the mean ± SD of three independent experiments. Two-way ANOVA followed by Tukey's multiple-comparisons test: *n*=3. ****P* < 0.001.

(D)Dose–response survival curves of control or CYLD-knockdown PC-3 cells stably expressing indicated constructs and treated with increasing concentrations of Olaparib. Means and SDs are represented. One-way ANOVA with Tukey's correction for multiple comparisons; *n*=3. ns, non-significant; ****P* < 0.001.

(E and F) Colony formation assays of CYLD-knockdown PC-3 cells stably expressing indicated constructs. Representative images (E) and quantification (F). Data were presented as the mean ± SD of three independent experiments. Two-way ANOVA followed by Tukey's multiple-comparisons test; *n*=3. ns, non-significant; ***P < 0.001.

(G)Immunoblot analysis of CYLD and TIRR in whole-cell lysates from UM-UC-3 cells stably expressing indicated constructs.One-way ANOVA with Tukey's correction for multiple comparisons; *n*=3. ns, non-significant; ****P* < 0.001.

(H and I) Representative immunofluorescence images (H) and quantification of 53BP1 (I) foci in UM-UC-3 cells stably expressing indicated constructs, with IR (6 Gy). Scale bar, 10 µm. Data are presented as mean ± SD from 20 fields (>50 cells per condition) across three biological replicates. Two-way ANOVA followed by Tukey's multiple-comparisons test; ns, non-significant; ****P < 0.0001.

(J) Control or CYLD knockdown PC-3 cells were injected subcutaneously into SCID mice and treated with vehicle or Olaparib (50 mg/kg). Representative body weight data are shown as mean ± SD. Two-way ANOVA followed by Tukey's multiple-comparisons test. (*n* = 9 mice per group). ns, non-significant.

(K and L) Quantification of γ-H2AX number per field (K) and TIRR scores (L). Two-way ANOVA followed by Tukey's multiple-comparisons test. (*n* = 9 mice per group). *****P* < 0.0001.
